# SMRT sequencing of a full-length transcriptome reveals transcript variants involved in C18 unsaturated fatty acid biosynthesis and metabolism pathways at chilling temperature in *Pennisetum giganteum*

**DOI:** 10.1186/s12864-019-6441-3

**Published:** 2020-01-16

**Authors:** Qingyuan Li, Conglin Xiang, Lin Xu, Jinghua Cui, Shao Fu, Baolin Chen, Shoukun Yang, Pan Wang, Yanfeng Xie, Ming Wei, Zhanchang Wang

**Affiliations:** 1 Forestry and Fruit Tree Research Institute, Wuhan Academy of Agricultural Sciences, Wuhan, China; 2 College of Horticulture and Forestry Sciences, Huazhong Agricultural University, Wuhan, China

**Keywords:** *Pennisetum giganteum*, Full-length transcriptome, Alternative splicing, C18 unsaturated fatty acids, Chilling temperature

## Abstract

**Background:**

*Pennisetum giganteum*, an abundant, fast-growing perennial C_4_ grass that belongs to the genus *Pennisetum*, family Poaceae, has been developed as a source of biomass for mushroom cultivation and production, as a source of forage for cattle and sheep, and as a tool to remedy soil erosion. However, having a chilling-sensitive nature, *P. giganteum* seedlings need to be protected while overwintering in most temperate climate regions.

**Results:**

To elucidate the cold stress responses of *P. giganteum*, we carried out comprehensive full-length transcriptomes from leaf and root tissues under room temperature (RT) and chilling temperature (CT) using PacBio Iso-Seq long reads. We identified 196,124 and 140,766 full-length consensus transcripts in the RT and CT samples, respectively. We then systematically performed functional annotation, transcription factor identification, long non-coding RNAs (lncRNAs) prediction, and simple sequence repeat (SSR) analysis of those full-length transcriptomes. Isoform analysis revealed that alternative splicing events may be induced by cold stress in *P. giganteum*, and transcript variants may be involved in C18 unsaturated fatty acid biosynthesis and metabolism pathways at chilling temperature in *P. giganteum*. Furthermore, the fatty acid composition determination and gene expression level analysis supported that C18 unsaturated fatty acid biosynthesis and metabolism pathways may play roles during cold stress in *P. giganteum*.

**Conclusions:**

We provide the first comprehensive full-length transcriptomic resource for the abundant and fast-growing perennial grass *Pennisetum giganteum*. Our results provide a useful transcriptomic resource for exploring the biological pathways involved in the cold stress responses of *P. giganteum*.

## Background

Temperature is a major environmental factor that affects plant growth, development, productivity and distribution [1]. In agriculture, cold stress may limit production, causing preharvest and postharvest damage and resulting in qualitative and quantitative losses [2]. Plants from temperate regions can increase their freezing tolerance by being exposed to chilling and non-freezing temperatures, which is known as cold acclimation [3]. By contrast, plants of tropical and subtropical origins, such as rice, maize, and C_4_ grasses, largely lack such a capacity for cold acclimation and are sensitive to chilling stress [1].

As the principal barrier between the cytoplasm and the extracellular milieu, the plasma membrane is regarded as a key site of injury during cold stress [4]. As temperatures below the optimal requirements for organisms cause membrane lipid rigidity and abnormal cellular activities, plants respond to such environmental influences by remodeling the lipid composition of their membrane [5]. As phospholipids rich in unsaturated fatty acids often have a considerably lower transition temperature compared to phospholipids containing high amounts of saturated fatty acids, plants with higher unsaturated fatty acids content in the plasma membranes usually exhibit strong cold tolerance [6, 7]. The maintenance of polyunsaturated fatty acid levels in chloroplast lipids has been shown to contribute to survival at low temperatures and the normal formation of chloroplast membranes in plants under cold stress [8]. Trienoic fatty acids (TAs), such as hexadecatrienoic acid (16:3) and linolenic acid (18:3), which are considered to be the major polyunsaturated fatty acid species in plant membrane lipids, are important to ensure the correct biogenesis and maintenance of chloroplasts during plant growth under low temperatures [9]. A study on *Camellia japonica* showed that α-linolenic acid biosynthesis and metabolism pathways may play roles in plant cold responses [7].

C_4_ photosynthesis is theoretically more efficient than C3 photosynthesis in light, nitrogen and water use [10]. C_4_ grasses dominate most open biomes in tropical and subtropical areas, where they achieve greater biomass and higher growth rates [11]. However, in cooler environments, the peak yields of most C_4_ plants are markedly reduced [12]. As a result, the present global distribution of C_4_ grasses is largely limited to warmer climate regions, and strong positive relationships between C_4_ grass abundance and growing season temperature have been documented at continental scales and along elevational gradients on tropical mountains across the globe [13].

*Pennisetum giganteum*, an abundant, fast growing perennial C_4_ grass that belongs to the genus *Pennisetum*, family Poaceae, is native to eastern and northeastern African tropical regions, such as Kenya, Eritrea and Ethiopia. This grass has been planted in more than 30 provinces in China and more than 80 countries worldwide [14]. In addition, at present, *P. giganteum* has been developed as a source of biomass for mushroom cultivation and production, a source of forage for cattle and sheep, and a tool to remedy soil erosion [15–18]. However, having a chilling-sensitive nature, *P. giganteum* seedlings need to be protected during overwintering in most temperate climate regions. Therefore, improving the chilling tolerance of *P. giganteum* will be important for livestock husbandry and earth ecology.

Third-generation sequencing platforms, such as single-molecule real-time (SMRT) sequencing from PacBio, can generate full-length cDNA sequences without assembly [19, 20]. Isoform sequencing (Iso-Seq), which is based on the SMRT sequencing platform, has been used to analyze full-length transcriptomes in various plant species [21, 22]. In this study, we used PacBio Iso-Seq to generate comprehensive full-length transcriptomes for *P. giganteum* under room temperature (RT) and chilling temperature (CT). We then systematically carried out functional annotation, transcription factor identification, long non-coding RNAs (lncRNAs) prediction, and simple sequence repeat (SSR) analysis of those full-length transcriptomes. Moreover, isoform analysis revealed the complexity of alternative splicing in *P. giganteum*, and transcript variants may be involved in C18 unsaturated fatty acid biosynthesis and metabolism pathways at chilling temperature in *P. giganteum*. In this study, we not only systematically characterized the profile of the *P. giganteum* full-length transcriptome but also provided a valuable resource for investigating the biological pathways involved in the cold response in *P. giganteum*.

## Results

### *P. giganteum* transcriptome analysis using PacBio Iso-seq

Two pooled samples (from leaf and root tissues) of RT and CT were sequenced to obtain a wide coverage of the *P. giganteum* full-length transcriptome using PacBio Iso-Seq. For the RT sample, a total of 509,371 circular consensus sequencing (CCS) reads were generated, with a total of 558,634,435 nucleotides. For the CT sample, a total of 371,590 CCS reads were generated, with a total of 442,366,361 nucleotides (Additional file 4: Table S1). The subreads distribution is shown in Additional file 1: Figure S1a.

By applying the standard Iso-Seq classification and clustering protocol on the above CCS reads, we produced 393,678 full length reads, including 382,945 full-length non-chimeric (Flnc) reads with an average length of 2370 bp for the RT sample. For the CT sample, we generated 273,168 full length reads, including 263,302 Flnc reads with an average length of 2489 bp. Finally, 196,124 and 140,766 full-length consensus transcripts were generated in the RT and CT samples, respectively (Table 1). The Flnc reads distribution is shown in Additional file 1: Figure S1b.

**Table 1:** Summary of consensus transcripts after Iso-Seq classification and clustering protocol

Sample	Number of 5′-primer reads	Number of 3′-primer reads	Number of Poly-A reads	Number of full length reads	Number of flnc reads	Average flnc read length (bp)	Full-length percentage (%)	Consensus transcripts
RT	464,021	464,131	448,301	393,678	382,945	2370	77	196,124
CT	330,088	331,916	324,607	273,168	263,302	2489	74	140,766

After combining the RT and CT data, a total of 336,890 transcripts and 319,926 unigenes were generated, and the distribution of transcripts and unigenes is shown in Additional file 5: Table S2.

### Functional annotation of the assembled transcriptome

To obtain a comprehensive functional annotation of the *P. giganteum* transcriptome, we assessed the non-redundant transcripts from RT and CT samples using a BLASTX search against the following databases: Nr (NCBI non-redundant protein sequences), Nt (NCBI non-redundant nucleotide sequences), Swiss-Prot (a manually annotated and reviewed protein sequence database), GO (Gene Ontology), COG (cluster of orthologous groups) and KEGG (Kyoto Encyclopedia of Genes and Genomes). For the RT sample, 163,748 (83.49%), 165,942 (84.61%), 124,098 (63.28%), 65,341 (33.32%), 87,244 (44.48%), and 158,795 (80.97%) unigenes returned BLAST results and showed identity with sequences in the Nr, Nt, SwissProt, GO, KOG and KEGG databases, respectively. For the CT sample, 120,529 (85.62%), 122,147 (86.77%), 99,970 (71.02%), 71,041 (50.47%), 73,324 (52.09%), 117,980 (83.81%) unigenes returned BLAST results and showed identity with sequences in the Nr, Nt, SwissProt, GO, COG and KEGG databases, respectively (Additional file 6: Table S3).

**Fig. 1:**
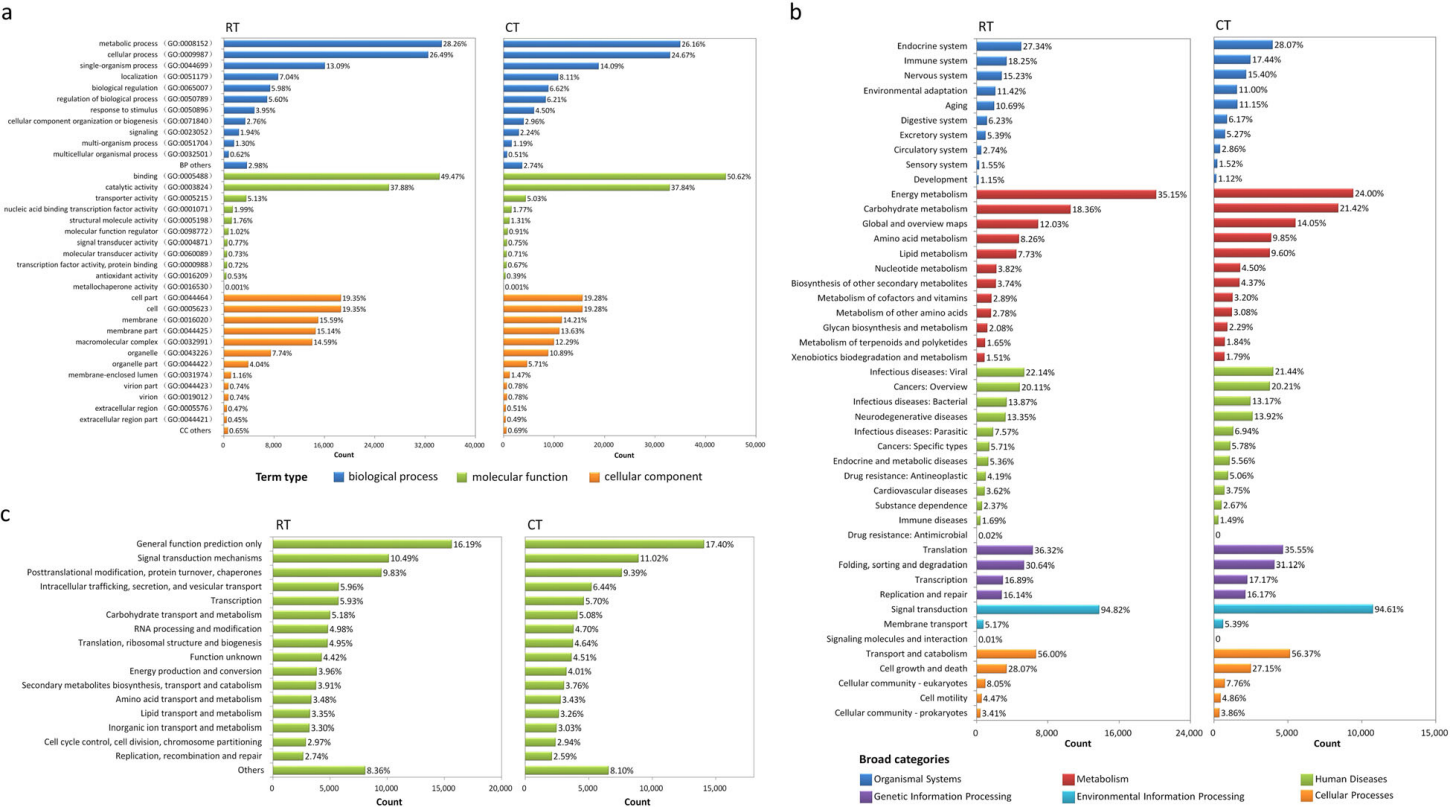
Function annotation and classification of *P. giganteum* assembled transcriptomes under RT and CT. **a** GO classification of the annotated unigenes in RT and CT samples. **b** KEGG classification of the annotated unigenes in RT and CT samples. **c** COG classification of the putative proteins in RT and CT samples

To determine the potential functions of unigenes, we used GO assignments to classify the predicted *P. giganteum* genes. It is interesting that unigenes identified from the GO database in the CT sample (71,041) were more than those in the RT sample (65,341), although the total unigenes in the CT sample (140,766) were less than those in the RT sample (196,124). Overall, we observed highly similar GO functional classifications between the RT and CT samples (Fig. 1a). In terms of biological processes, ‘metabolic processes’ (28.26% in RT sample and 26.16% in CT sample) and ‘cellular processes’ (26.49% in RT sample and 24.67% in CT sample) were the top two GO terms in both treatments. It is notable that the percentages of unigenes in the terms ‘biological regulation’, ‘regulation of biological process’, ‘response to stimulus’ and ‘signaling’ in the CT sample were greater than that of the RT sample, which may indicate that plants were undergoing stress stimulated biological regulations in the CT sample. In the molecular function category, the unigenes were predominantly assigned to the ‘binding’ (49.47% in RT sample and 50.62% in CT sample) and ‘catalytic activities’ (37.88% in RT sample and 37.84% in CT sample) groups in both treatments. In the cellular component category, the unigenes were frequently assigned to ‘cell part’ (~ 20%), ‘cell’ (~ 20%), ‘membrane’ (~ 15%) in both CT and RT samples.

To identify the biological pathways in the annotated *P. giganteum* sequences, we annotated the unigenes to the reference pathways in the KEGG using KeggArray software. A total of 158,795 and 117,980 unigenes were assigned to six specific pathways, including ‘Organism Systems’, ‘Metabolism’, ‘Human disease’, ‘Genetic Information Processing’, ‘Environmental Information Processing’, and ‘Cellular Processes’ in the RT and CT samples, respectively (Fig. 1b). As with the GO classification, the percentages of different classes of KEGG pathway terms were highly similar in the RT and CT samples. Nearly 40% of annotated unigenes were classified as ‘Metabolism’-related pathways, in which ‘Energy metabolism’, ‘Carbohydrate metabolism’, and ‘Global and overview maps’ were the top 3 pathways with the most abundant unigenes in both RT and CT samples. The difference between the two treatments with respect to KEGG annotation is the percentages of genes involved in ‘Energy metabolism’ and ‘Carbohydrate metabolism’. There was a higher percentage of unigenes associated with ‘Energy metabolism’ in RT compared with CT sample (35.15% compared with 24.00%); however, there was a lower percentage of unigenes associated with ‘Carbohydrate metabolism’ in RT compared with CT sample (18.36% compared with 21.42%) (Fig. 1b).

To classify the orthologous gene products, 87,244 and 73,324 unigenes were subdivided into COG classifications in the RT and CT samples, respectively (Fig. 1c). The percentages of different classes of COG classifications were highly similar in the RT and CT samples. In both treatments, the cluster of ‘general function prediction only’ (16.19% in RT sample and 17.40% in CT sample) represented the largest group, followed by ‘signal transduction mechanisms’ (10.49% in RT sample and 11.02% in CT sample) and ‘posttranslational modification, protein turnover, chaperones’ (9.83% in the RT sample and 9.39% in the CT sample).

Taken together, the results from GO, KEGG and COG annotation and classification of unigenes allowed us to obtain a comprehensive functional characterization for the full-length transcriptomes from CT and RT treatments of *P. giganteum*. The overall similar functional classification of transcripts in these two treatments indicates that the transcriptome at the pathway level is generally conserved, although some subtle differences can be found between the CT and RT samples.

### Transcription factors identification

**Fig. 2:**
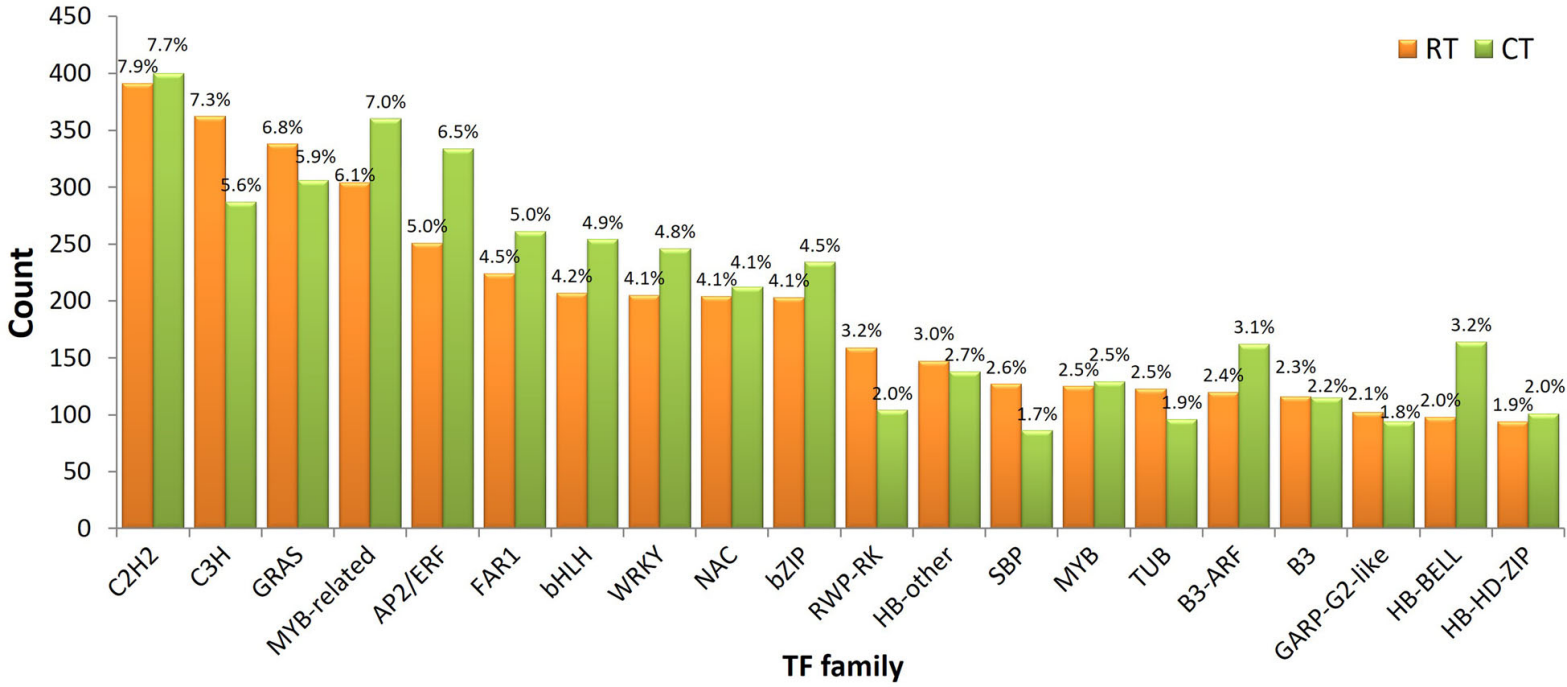
Putative TF gene families in *P. giganteum* assembled transcriptomes under RT and CT

A total of 4974 and 5170 putative TF genes were identified in the RT and CT samples, respectively (Additional file 7: Table S4). Notably, the number of putative TF genes in the CT sample was greater than that in the RT sample, although the total number of unigenes in the CT sample (140,766) was less than that in the RT sample (196,124). Among all TF families, the C2H2 family was the largest group in both RT and CT samples (391, 7.86% in RT sample and 400, 7.74% in CT sample). The C3H family (362, 7.28%) and the GRAS family (338, 6.80%) were followed by in the RT sample. For the CT sample, the second and third largest groups were represented by the MYB-related family (360, 6.96%) and the AP2/ERF family (334, 6.46%). Furthermore, the unigenes in the FAR1, bHLH, WRKY, bZIP, B3-ARF and HB-BELL TF families in the CT sample were more than those in the RT sample (Fig. 2, Additional file 7: Table S4).

### LncRNA prediction

**Fig. 3:**
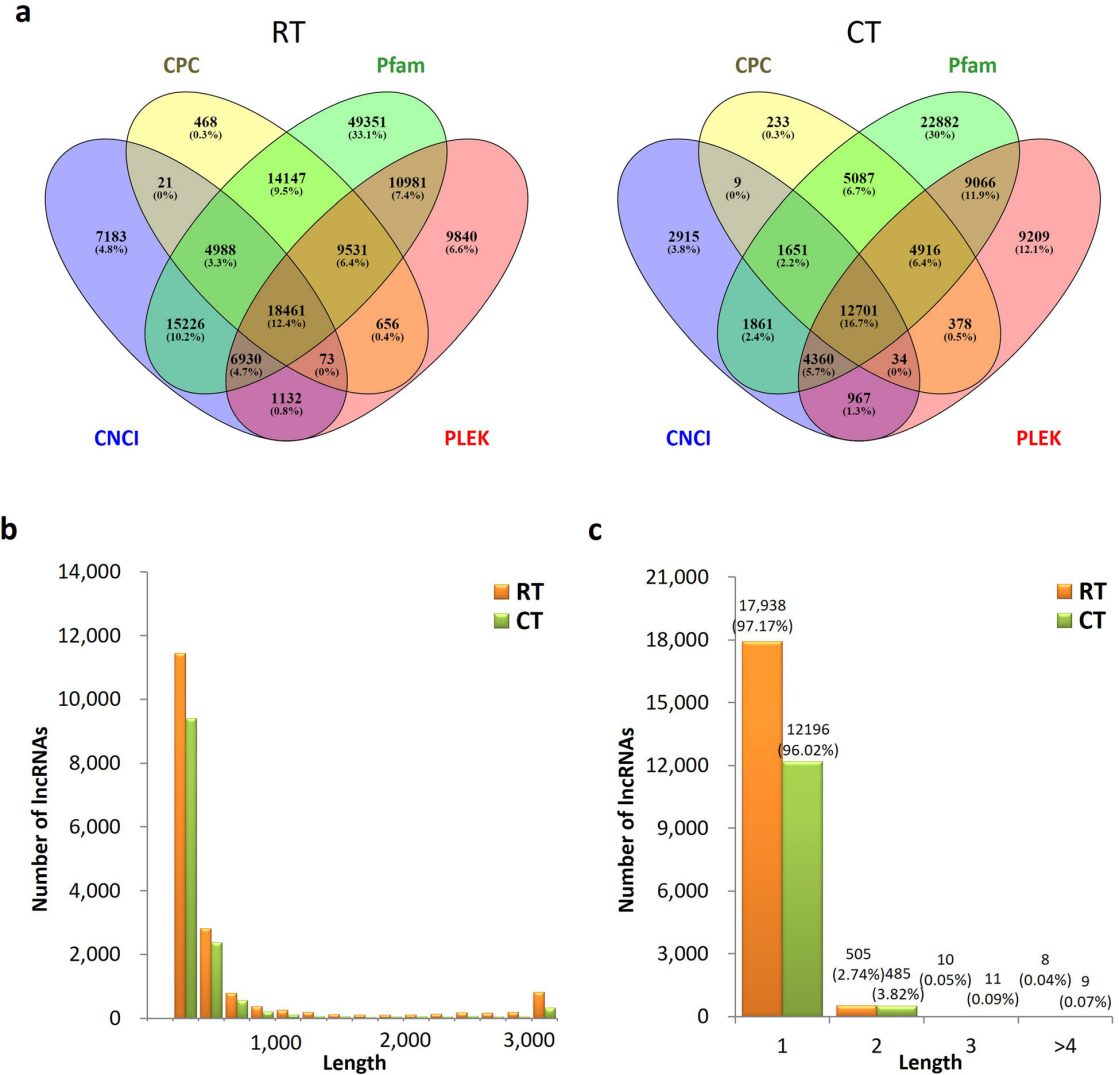
Identification of *P. giganteum* lncRNAs. **a** Venn diagram of the number of lncRNAs predicted by CNCI, CPC, Pfam and PLEK. **b** Length distribution of identified lncRNAs in RT and CT samples. **c** Distribution of isoform numbers for lncRNAs in RT and CT samples

LncRNAs from these PacBio Iso-Seq data sets were predicted by CNCI, Pfam, PLEK and CPC protein structure domain analysis. In total, 18,461 and 12,701 candidate lncRNAs of ≥200 bp were predicted by all four methods in the RT and CT samples, respectively (Fig. 3a). The lncRNAs had a length ranging from 200 to 15,913 bp with an average length of 559 bp in the RT sample and a length ranging from 200 to 7359 bp with an average length of 491 bp in the CT sample (Additional file 8: Table S5). The length distribution of lncRNAs is shown in Fig. 3b, and most of them were single-isoform transcripts present in both treatments (Fig. 3c). The functions of these lncRNAs need to be further characterized.

### Genic-SSR identification

SSRs were highly abundant in the assembled *P. giganteum* transcriptome. In total, 39,309 potential SSRs with a minimum of five repetitions for all motifs (di- to hexa- nucleotides) were identified from 33,463 contigs, representing 12.29% of the total 319,926 unigenes in RT and CT combined transcriptome. The frequency of occurrence of SSR loci was one in every 17.6 kb of full-length unigene sequences. Among all repeat types, the length of SSRs was distributed from 12 to 140 bp with an average of 16.61 bp.

**Fig. 4:**
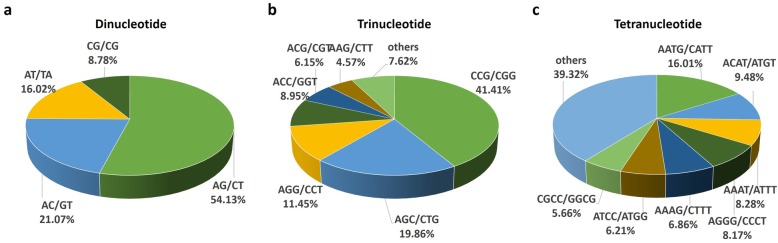
Percentages of different motifs among dinucleotide (**a**), trinucleotide (**b**) and tetranucleotide (**c**) repeats in *P. giganteum* RT and CT combined transcriptome

Incidences of different repeat types and frequencies for each motif were evaluated based on the repeat unit number (Table 2). SSRs existed mainly as dinucleotide repeats and trinucleotide repeats, accounting for 97.65%. Trinucleotide repeats, comprising 72.11% of the total SSRs, were the most abundant repeat unit, followed by di- (24.37%), tetra-(2.34%), penta-(0.62%) and hexa-nucleotides (0.56%). Most (97.39%) of the motifs had 5–10 repeat units, while motifs with more than 10 reiterations were rare, exhibiting a frequency of 2.61%. Within the identified SSRs, AG/CT comprised 54.13% of all dinucleotide repeat motifs and was the most common type (Fig. 4a). The predominant trinucleotide repeat motifs were CCG/CGG and AGC/CTG, which accounted for 41.41 and 19.86%, respectively (Fig. 4b). In tetranucleotide repeats, the most frequent motif was AATG/CATT (16.01%) followed by ACAT/ATGT (9.48%) and AAAT/ATTT (8.28%) (Fig. 4c).

**Table 2:** Frequencies of different SSR repeat motif types observed in *P. giganteum* transcriptome

SSR motif	Repeat number								Percentage
	5	6	7	8	9	10	> 10	Tatal	(%)
Dinucleotide	0	4596	1813	1216	710	387	859	9581	24.37
Trinucleotide	18,897	6009	2135	819	274	89	121	28,344	72.11
Tetranucleotide	649	134	85	26	9	2	13	918	2.34
Pentanucleotide	170	28	18	6	2	2	19	245	0.62
Hexanucleotide	127	54	17	4	2	2	15	221	0.56
Total	19,843	10,821	4068	2071	997	482	1027	39,309	100.00
Percentage (%)	50.48	27.53	10.35	5.27	2.54	1.23	2.61	100.00

### Gene alternative splicing detection

**Fig. 5:**
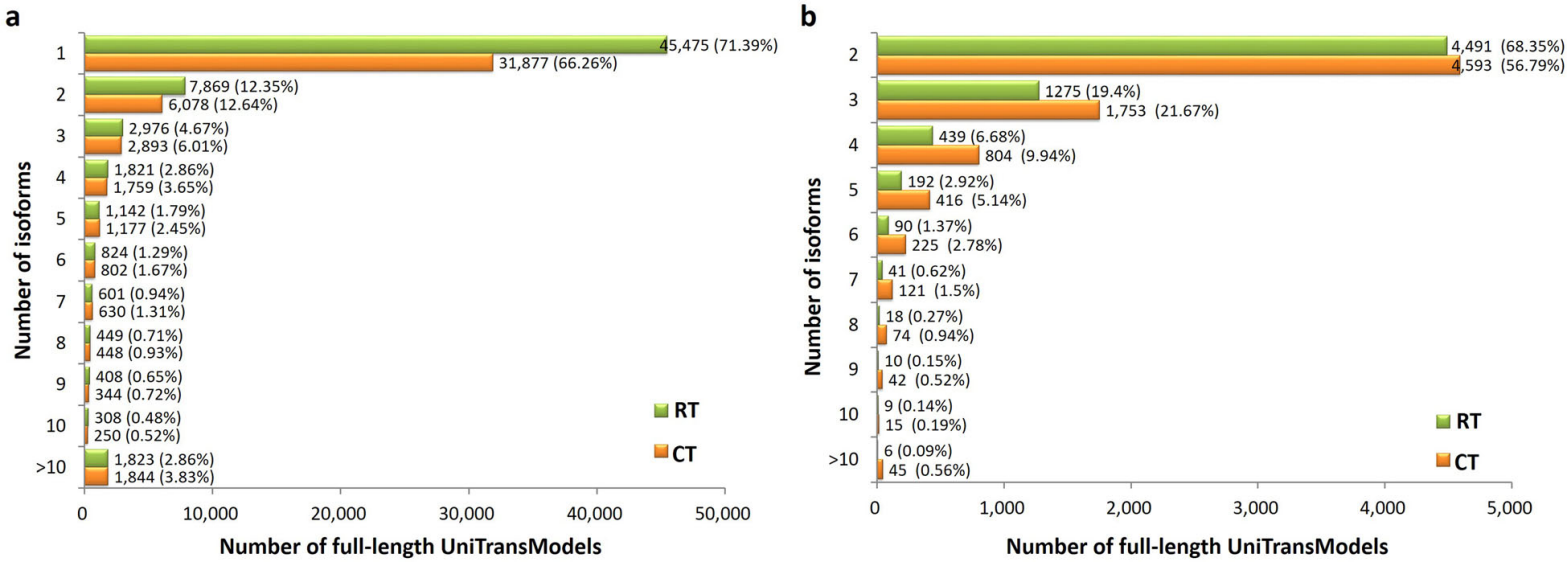
Isoform analysis of *P. giganteum* full-length transcriptomes using Iso-Seq. **a** Distribution of isoform numbers for UniTransModels in RT and CT samples. **b** Distribution of isoform numbers for UniTransModels that have alternative splicing events in both samples

One advantage of PacBio Iso-Seq is its ability to describe the complexity of alternative splicing at the whole-transcriptome scale. To detect the alternative splicing event in the *P. giganteum* transcriptome, the Coding GENome reconstruction Tool (Cogent) was used to further partition these error-corrected non-redundant transcripts into transcript families and reconstruct each family into one or several full-length unique transcript model(s) (referred to as UniTransModel). In total, 63,696 and 48,102 full-length UniTransModels were obtained for RT and CT samples, respectively. Then, transcript isoforms were identified in both samples, a total of 28.61% of full-length UniTransModels had more than one isoform in the RT sample and a slightly more percentage (33.74%) in the CT sample (Fig. 5a).

We identified alternative splicing events by using the UniTransModels as our reference. In total, 6571 and 8088 UniTransModel-based alternative splicing events were identified in RT and CT samples, representing 10.32% of the 63,696 full-length UniTransModels for RT sample and 16.81% of the 48,102 full-length UniTransModels for CT sample, respectively. The transcript isoform numbers in both samples with alternative splicing events are shown in Fig. 5b. It is notable that the percentages of UniTransModels had two isoforms and more in CT sample were all higher than that in the RT sample. These results indicated that alternative splicing events may be induced by cold stress in *P. giganteum*.

By mapping Illumina short reads to transcript models, we were able to confirm the reliability of isoform detection using our pipeline, even in the absence of a reference genome (Additional file 2: Figure S2). We also detected different splicing isoforms of the same UniTransModels in RT and CT samples (Additional file 2: Figure S2).

### Gene alternative splicing involved in α-linolenic acid biosynthesis and metabolism pathways

**Fig. 6:**
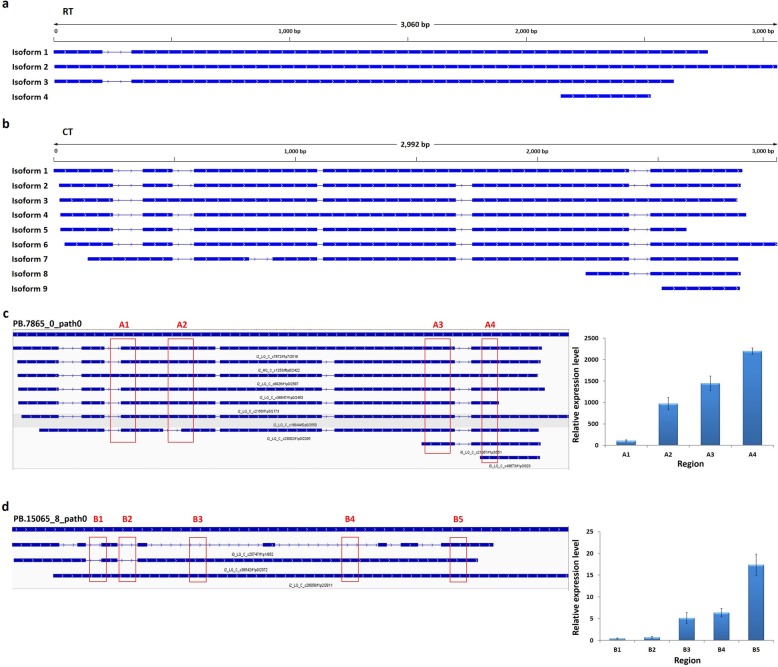
Gene alternative splicing involved in α-linolenic acid biosynthesis and metabolism pathways. Different isoforms of the *MFP2* gene in *P. giganteum* in RT (**a**) and CT (**b**) samples. For each isoform, blocks in blue represent exons and lines in- between represent introns. Validation of AS events in PB.7865_0_path0 (**c**) and PB.15065_8_path0 (**d**) using qRT-PCR. Different primer pairs were designed to analysis the relative transcript levels of different regions which included in different isoforms. Data in (**c**, **d**) are means ± SE from three biological independent experiments

The octadeca-carbon unsaturated fatty acids play important roles in plant cold responses. We searched our UniTransModels with alternative splicing events using BLASTX against the KEGG databases. We determined that 12 genes involved in the α-linolenic acid metabolism pathway had alternative splicing events in the RT sample and 14 in the CT sample (Additional file 9: Table S6). We also found that several of these genes might have different transcription isoforms in RT and CT samples. For example, the enoyl-CoA hydratase/3-hydroxyacyl-CoA dehydrogenase gene *MFP2* had 4 isoforms in the RT sample whereas had 9 isoforms in the CT sample (Fig. 6a and b).

Interestingly, we also determined that 6 genes involved in α-linolenic acid biosynthesis pathway had alternative splicing events in the CT sample, including one very-long-chain (3R)-3-hydroxyacyl-CoA dehydratase (*HACD*) gene, PB.15065_8_path0, one acyl-[acyl-carrier-protein] desaturase (*FAB2*) gene, PB.11646_0_path0, three 3-oxoacyl-[acyl-carrier protein] reductase (*FabG*) genes, PB.14731_0_path0, PB.18504_0_path0, PB.3134_0_path0, and one acyl-coenzyme A thioesterase (*ACOT*) gene, PB.8528_0_path0 (Additional file 9: Table S6), whereas no genes were found that had alternative splicing events in such a pathway in the RT sample.

Furthermore, qRT-PCR were conducted to validate the AS events involved in α-linolenic acid biosynthesis and metabolism pathways. Different primer pairs were designed to analysis the relative transcript levels of different regions which included in different isoforms. In PB.7865_0_path0, region A1, A2, A3 and A4 were included in 1, 6, 8 and 9 isoforms, respectively (Fig. 6c, left panel). The result of transcript level analysis showed that the more isoforms contained in the region, the higher relative transcript level it had (Fig. 6c, right panel). In PB.15065_8_path0, region B1, B2, B3, B4 and B5 were included in 1, 1, 2, 2 and 3 isoforms, respectively (Fig. 6d, left panel). The result showed that the region B5 had the highest relative transcript level, followed by B3 and B4, then B1 and B2 (Fig. 6d, right panel).

These results indicated that the alternative splicing events in α-linolenic acid biosynthesis and metabolism pathway genes may be induced by low temperature in *P. giganteum*. Our full-length Iso-Seq transcriptome can provide not only additional information for characterization of the α-linolenic acid biosynthesis and metabolism biosynthesis pathways at a deeper transcription isoform level but will also help understand other biological pathways under normal conditions and cold stress in *P. giganteum*.

### C18 unsaturated fatty acid contents were enhanced in *P. giganteum* leaves under cold stress

**Fig. 7:**
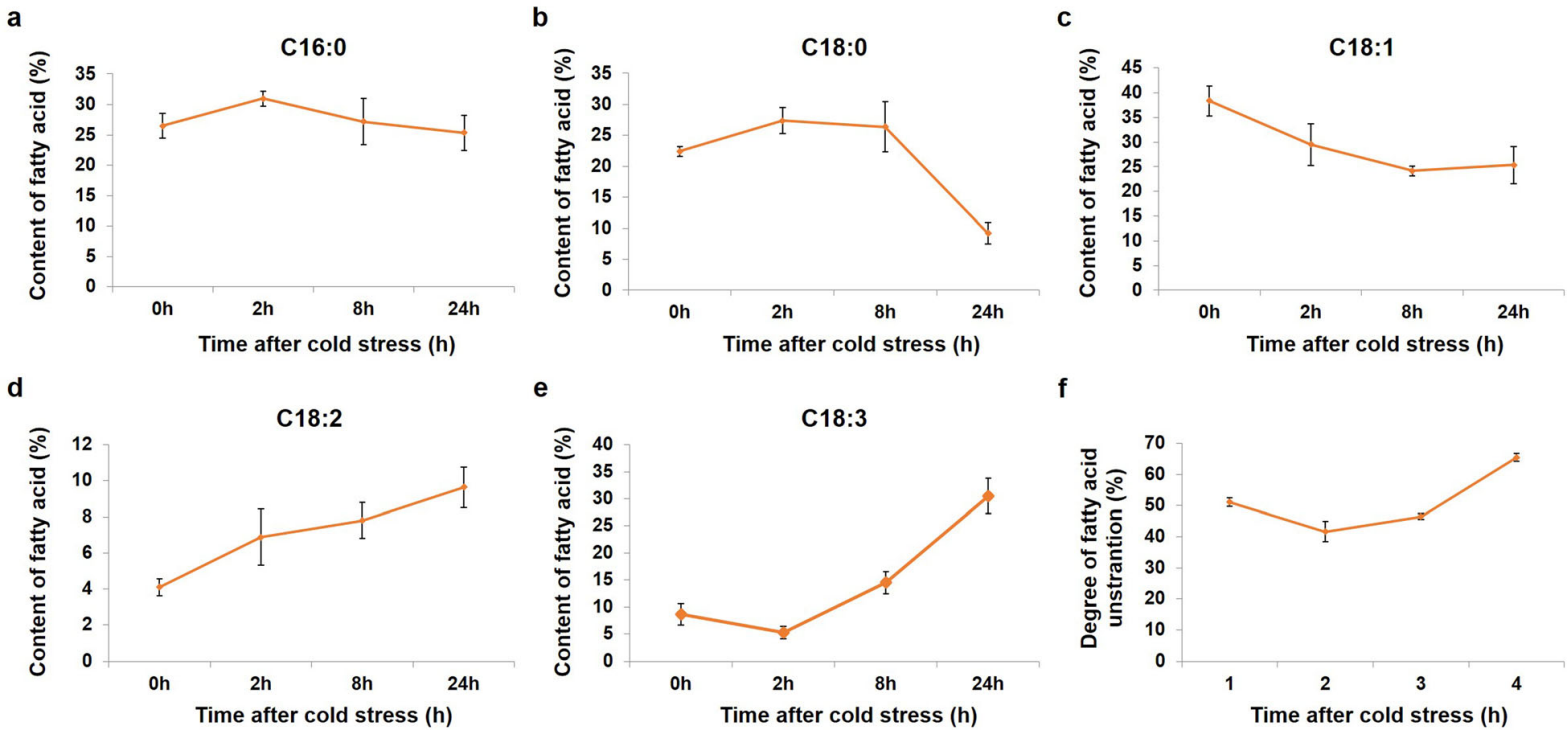
Fatty acid contents in *P. giganteum* leaves under cold stress. The content of palmitic acid (16:0) (**a**), stearic acid (18:0) (**b**), oleic acid C18:1 (**c**) linoleic acid C18:2 (**d**) and α-linolenic acid C18:3 (**e**) are expressed in percentage. The degree of fatty acid unsaturation (**f**) were presented as the percentage of unsaturated fatty acid in the total fatty acid. Data reported are mean valuse of three independent experiments ± SE

To confirm that the unsaturated fatty acid biosynthesis pathway is important for the cold stress response in *P. giganteum*, we determinated the fatty acid compositions in *P. giganteum* leaves during cold stress using gas chromatography. As shown in Fig. 7, the content of palmitic acid (16:0), stearic acid (18:0) and oleic acid (18:1) gradually decreased during cold stress, while the content of linoleic acid (18:2) and α-linolenic acid (18:3) increased significantly during the same process (Fig. 7a–e). Moreover, the degree of fatty acid unsaturation in *P. giganteum* leaves gradually increased during cold stress (Fig. 7f), which was consistent with the results from the determination of fatty acid composition. These results indicated that the unsaturated fatty acid biosynthesis pathway was activated in the cold stress response in *P. giganteum*.

### Genes in α-linolenic biosynthesis and metabolism pathways were affected at chilling temperature

To further study the pathways that involved in cold stress response in *P. giganteum*, the differentially expressed genes (DEGs) between cold-stressed and non-stressed samples were determined. In total, 17,134, 26,266 and 19,908 DEGs were identified at 2, 8 and 24 h of cold stress, respectively. Among all DEGs, 11,500 were present at all three sampling time points, and 1874, 4583 and 6283 were specific for 2, 8 and 24 h of cold stress, respectively (Additional file 3: Figure S3).

**Fig. 8:**
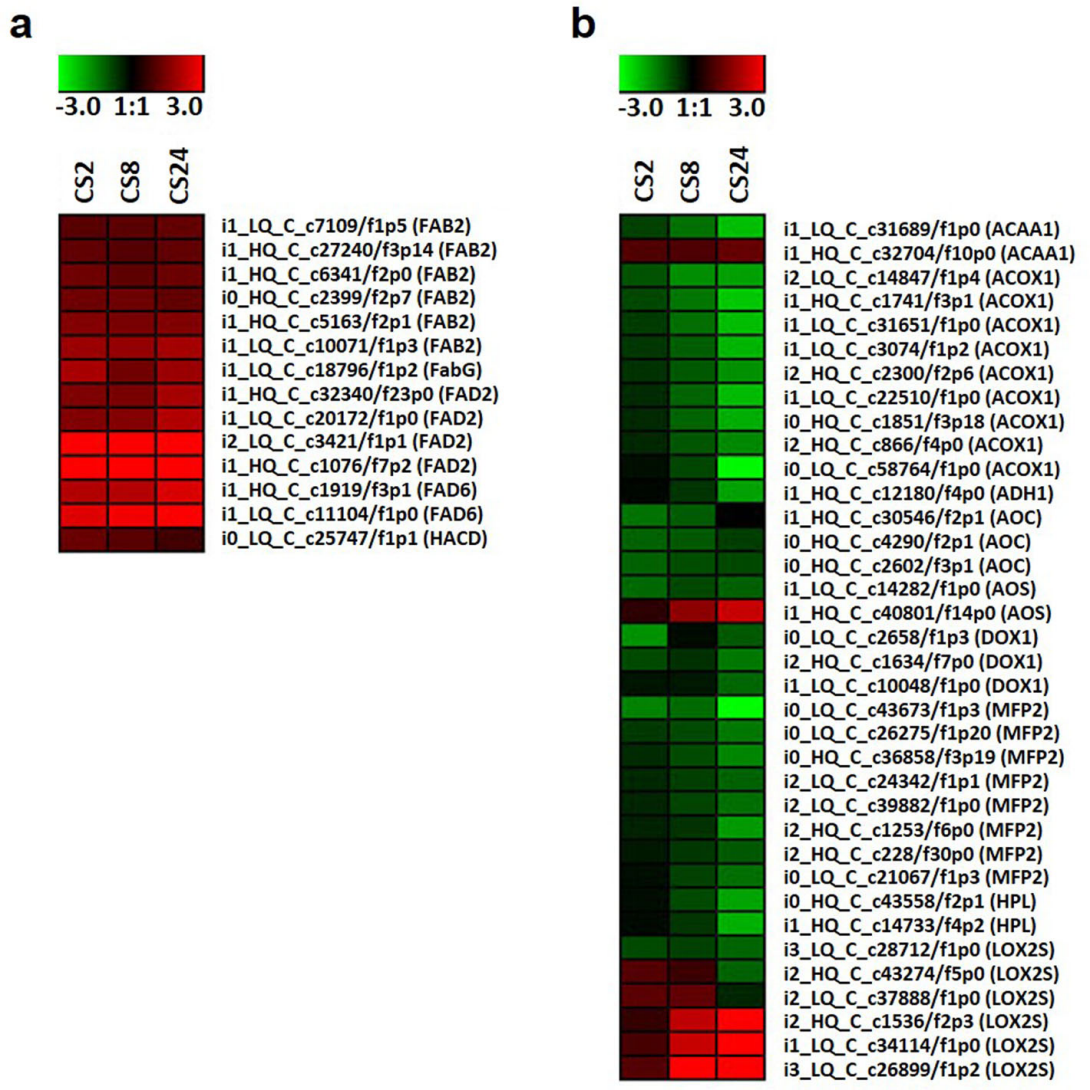
α-linolenic biosynthesis and metabolism Pathways genes in cold stress responses in *P. giganteum*. **a** α-linolenic biosynthesis pathway; **b** α-linolenic metabolism pathway

As C18 unsaturated fatty acid contents were enhanced by cold stress in *P. giganteum*, we identified the DEGs involved in α-linolenic fatty acid biosynthesis and metabolism pathways. Several α-linolenic fatty acid biosynthesis genes were differentially expressed during cold stress as determined by RNA-seq analysis, including six acyl-[acyl-carrier-protein] desaturase (*FAB2*) genes, one 3-oxoacyl-[acyl-carrier protein] reductase (*FabG*) genes, four omega-6 fatty acid desaturase (*FAD2*) genes, two acyl-lipid omega-6 desaturase (*FAD6*) genes and one very-long-chain (3R)-3-hydroxyacyl-CoA dehydratase (*HACD*) gene (Fig. 8a). It is notable that all these genes were up-regulated during cold stress. This result indicated that the α-linolenic fatty acid biosynthesis pathway was activated during cold stress in *P. giganteum*. In α-linolenic fatty acid metabolism pathway, 36 genes were differentially expressed during cold stress, including two acetyl-CoA acyltransferase 1 (*ACAA1*) genes, nine acyl-CoA oxidase (*ACOX1*) genes, one alcohol dehydrogenase class-P (*ADH1*) gene, three allele oxide cyclase (*AOC*) genes, two hydroperoxide dehydratase (*AOS*) genes, two alpha-dioxygenase (*DOX1*) genes, eight enoyl-CoA hydratase/3-hydroxyacyl-CoA dehydrogenase (*MFP2*) genes, four hydroperoxide lyase (*HPL*) genes and six lipoxygenase (*LOX2S*) genes (Fig. 8b). It is interesting that genes involved in α-linolenic fatty acid metabolism pathway were largely down-regulated during cold stress. This result suggested that the α-linolenic fatty acid metabolism pathway was inhibited during cold stress in *P. giganteum*.

These results indicated that the α-linolenic acid biosynthesis and metabolism pathway genes may be play important roles in cold stress responses in *P. giganteum*.

## Discussion

C_4_ plants play a key role in world agriculture, crops such as maize and sorghum are major contributors to world food production in both developed and developing nations, and C_4_ grasses are the major plant sources of bioenergy [23]. However, most C_4_ plants are chilling sensitive [12]. It is important to develop an effective strategy for the genetic breeding of chilling tolerance in *P. giganteum*.

With the application of third-generation sequencing technology that can produce average read lengths of more than 10,000 bp [24], transcriptome reconstruction and annotation, particularly for species without a reference genome sequence, has improved significantly. Although several transcriptomic analyses in the genus *Pennisetum* have been reported [25, 26], transcriptomic studies in *P. giganteum* are still rare. In this study, we generated the *P. giganteum* full-length transcriptomes from leaf and root tissues using PacBio Iso-Seq long reads. These large-scale and high-quality full-length transcriptome data not only provide useful reference data but can also be used to elucidate chilling response pathways and conduct subsequent functional genomics studies in *P. giganteum* and other plants of the genus *Pennisetum*. To the best of our knowledge, this report describes the first full-length transcriptomic analysis of *P. giganteum*. Moreover, a total of 178,330 of the 196,124 unigenes (90.93%) in the RT sample and 129,746 of the 140,766 unigenes (92.17%) in the CT sample were annotated to public databases (Nr, Nt, SwissProt, GO, KOG and KEGG) for comprehensive analysis (Additional file 6: Table S3). Compared with previous studies in plants of the genus *Pennisetum*, which reported 103,454 annotated unigenes from 197,466 unigenes [27], the present study obtained more complete annotation information.

LncRNAs are a large and diverse class of transcribed RNA molecules with a length of more than 200 nucleotides that do not encode proteins (or lack > 100 amino acid open reading frame) and potentially play important roles in the gene regulation of eukaryotic cells [28]. Despite accumulating evidence suggesting that lncRNAs have crucial roles in gene expression control during both developmental and differentiation processes, such as in dosage compensation, genomic imprinting, cell differentiation and organogenesis [29], only a relatively small proportion of lncRNAs have been studied in detail. In this study, lncRNAs were identified in both RT and CT samples (Fig. 3). Although the functions of these lncRNAs have not been characterized yet, these still provide a useful resource for understanding the potential functions of lncRNAs in *P. giganteum*.

In eukaryotic cells, approximately 95% of genes undergo RNA transcript splicing, where most introns are removed and exons are retained, resulting in multiple alternative transcripts (isoforms) of the gene(s) [30, 31]. In metazoans, alternative splicing plays an important role in generating different protein products that function in diverse cellular processes, including cell growth, differentiation and death [32]. In this study, UniTransModel-based alternative splicing events were found in both in RT and CT samples (Fig. 5). The high proportion of transcripts with multiple splicing isoforms suggests a high degree of transcriptome complexity in *P. giganteum*. Previous studies have suggested that alternative splicing may play important roles in plant adaptation to environmental stresses [33, 34]. A recent study showed that rapid and dynamic alternative splicing impacts the cold response in Arabidopsis [1]. Similarly, in this study, we found that the alternative splicing level in the CT sample was higher than that in the RT sample (Fig. 5), indicating that alternative splicing may be one of the major drivers of transcriptome reprogramming for the chilling response in *P. giganteum*.

Although no specific receptor has been identified in plants in response to cold stress, it has been considered that the plasma membrane itself could be the primary sensor of temperature fluctuations [35]. Unsaturated fatty acids constitute the major components of membrane lipids of both prokaryotes and eukaryotic organisms [5]. Previous studies showed that fatty acid compositions were altered during plants exposed to cold temperatures [36, 37], and the level of unsaturated fatty acids is associated with cold tolerance [6, 7]. In our study, transcript variants were involved in C18 unsaturated fatty acid biosynthesis pathway (Additional file 9: Table S6), and C18 unsaturated fatty acid contents were enhanced by cold stress (Fig. 7). These results imply that the C18 unsaturated fatty acid biosynthesis pathway might be involved in the chilling stress in *P. giganteum*.

It is notable that α-linolenic acid, one of the most important C18 unsaturated fatty acids in plants, is the substrate of jasmonic acid (JA). Genes involved in the α-linolenic acid metabolism pathway are also involved in JA biosynthesis [38, 39]. It is well known that JA and its derivatives are important regulators of plant responses to abiotic stresses [39, 40]. In the present study, we found that a number of genes involved in the α-linolenic acid metabolism pathway had alternative splicing events in both RT and CT samples, and several of these genes had different transcript isoforms in RT and CT samples (Fig. 6; Additional file 9: Table S6). More interestingly, in our gene expression analysis, all the DEGs involved in α-linolenic fatty acid biosynthesis pathway were up-regulated during cold stress; instead, the DEGs involved in α-linolenic fatty acid metabolism pathway were largely down-regulated during the same process (Fig. 8). These results indicate that as a downstream pathway of α-linolenic acid biosynthesis, the α-linolenic acid metabolism pathway may also be involved in chilling responses in *P. giganteum*. However, the relationship between the alternative splicing events and transcription levels of related genes, and the actual functions of the alternative splicing isoforms will need to be determined in future studies.

## Conclusions

In this study, we performed an in-depth full-length transcriptome analysis of the responses to chilling temperature in *P. giganteum*. Our results systematically characterized the transcriptome information of *P. giganteum* at RT and CT and revealed that transcript variants may be involved in C18 unsaturated fatty acid biosynthesis and metabolism pathways at chilling temperature in *P. giganteum*, further expanding our knowledge of chilling responses in C_4_ grasses.

## Methods

### Plant materials and preparation

Mother plant of *P. giganteum* seedlings were obtained from National Engineering Research Center of JUNCAO Technology, Fujian, China. The plant material was identified by Professor Shoukun Yang, Forestry and Fruit Tree Research Institute, Wuhan Academy of Agricultural Sciences. And the germplasm (No. WH-H-JJC-2013) is deposited in the Germplasm Resources Nursery of Forestry and Fruit Tree Research Institute, Wuhan Academy of Agricultural Sciences, Wuhan, China.

In this study, *P. giganteum* seedlings were grown in growth chambers in Forestry and Fruit Tree Research Institute, Wuhan Academy of Agricultural Sciences, China, under long-day conditions (16 h light/8 h dark) under white fluorescent light at 25 °C during the day and 22 °C at night. Plants were then divided into two groups. The first group served as the RT sample, while the other group was moved and cultured at 4 °C as the CT sample.

Leaves and roots of *P. giganteum* seedlings at RT and at 2, 8 and 24 h of CT were sampled for RNA isolation. Total RNA was extracted using a Plant Total RNA Extraction Kit (BioTeke, China) following the manufacturer’s instructions and then treated with RNase-free DNase I (Thermo Scientific, USA) to remove genomic DNA contamination. The quality of extracted RNAs was evaluated using an Agilent 2100 Bioanalyzer (SA Pathology, Adelaide, SA, Australia). For the RT sample, RNA isolated from leaves and roots was evenly pooled. For the CT sample, RNA isolated from leaves and roots at 2, 8 and 24 h of CT was evenly pooled.

### Library preparation and sequencing

RNA samples were sent to Beijing Novogene Bioinformatics Technology Co., Ltd., where the libraries were produced and sequenced. Briefly, the Iso-Seq library was prepared according to the Isoform Sequencing protocol (Iso-Seq) using the Clontech SMARTer PCR cDNA Synthesis Kit and the BluePippin Size Selection System protocol as described by Pacific Biosciences (PN 100–092–800-03).

### Data processing

Sequence data were processed using SMRT Link 4.0 software. Circular consensus sequence (CCS) was generated from subread BAM files, parameters: min_length 200, max_drop_fraction 0.8, no_polish TRUE, min_zscore − 999, min_passes 1, min_predicted_accuracy 0.8, and max_length 18,000. CCS. BAM files were output, which were then classified into full-length and non-full-length reads using pbclassify.py, ignorepolyA false, minSeqLength 200. Subsequently produced non-full-length and full-length fasta files were then fed into the cluster step, which performs isoform-level clustering (ICE) followed by final Arrow polishing, hq_quiver_min_accuracy 0.99, bin_by_primer false, bin_size_kb 1, qv_trim_5p 100, and qv_trim_3p 30.

### Gene functional annotation

Gene function was annotated based on the following databases: Nr (NCBI non-redundant protein sequences); Nt (NCBI non-redundant nucleotide sequences); KOG/COG (Clusters of Orthologous Groups of proteins); Swiss-Prot (A manually annotated and reviewed protein sequence database); KO (KEGG Ortholog database); and GO (Gene Ontology).

### Coding potential analysis and lncRNA identification

CNCI (Coding-Non-Coding-Index) profiles adjoining nucleotide triplets to effectively distinguish protein-coding and non-coding sequences independent of known annotations. We use CNCI with default parameters. CPC (Coding Potential Calculator) mainly assesses the extent and quality of the ORF in a transcript and searches the sequences with known protein sequence databases to clarify the coding and non-coding transcripts. We used the NCBI eukaryote protein database and set the e-value ‘1e-10’ in our analysis. We translated each transcript into all three possible frames and used Pfam Scan to identify the occurrence of any of the known protein family domains documented in the Pfam database. Any transcript with a Pfam hit would be excluded in the following steps. Pfam searches use default parameters of -E 0.001 -- domE 0.001. PLEK (Predictor of Long non-coding RNAs and Messenger RNAs based on an Improved k-mer Scheme) is an efficient alignment-free computational tool to distinguish lncRNAs from mRNAs in RNA-seq transcriptomes of species lacking reference genomes. PLEK is especially suitable for PacBio or 454 sequencing data and large-scale transcriptome data. We use PLEK with default parameters.

We filtered out the sequences with an ORF≥200 aa, after which the remaining sequences were characterized for potential non-coding RNAs, including small RNAs, microRNAs and lncRNAs.

### SSR analysis

SSR of the transcriptome was identified using MISA [41]. This analysis allows the identification and localization of perfect microsatellites, as well as compound microsatellites that are interrupted by a certain number of bases.

### Full-length unique transcript model reconstruction

The non-redundant transcripts were processed with Coding GENome reconstruction Tool (Cogent v1.4, https://github.com/Magdoll/Cogent). In general, Cogent first creates the k-mer profile of non-redundant transcripts and calculates pairwise distances and then clusters transcripts into families based on their k-mer similarity. Each transcript family is further reconstructed into one or several unique transcript model(s) (referred to as UniTransModels) using a De Bruijn graph method [21].

### Isoform identification

Error-corrected non-redundant transcripts (transcripts before Cogent reconstruction) were mapped to UniTransModels using GMAP v2014-08-04 [42]. Splicing junctions for transcripts mapped to the same UniTransModels were examined, and transcripts with the same splicing junctions were collapsed. Collapsed transcripts with different splicing junctions were identified as transcription isoforms of UniTransModels. AS events were detected with SUPPA using default settings [43].

### Fatty acid analysis

Leaves of *P. giganteum* seedlings at RT and at 2, 8 and 24 h of CT were sampled and used for lipid extraction and profiling. The fatty acid extraction was performed following a described protocol [44]. Briefly, samples of ~ 70 mg fresh weight heated at 90 °C in 2.5% (v/v) H2SO4 in methanol for 90 min in screw-capped tubes. After the addition of 500 μl of hexane containing 0.01% butylated hydroxytoluene, fatty acids were extracted into the organic phase by shaking and the tubes were centrifuged at low speed. The samples were analysed using an Agilent 7890 series gas chromatograph with a column (HP-INNOWax 19,091 N-133; 30 m6250 mm60.25 mm) and quantified with flame ionization detection.

### Gene expression quantification and differential expression analysi

To quantify transcript abundance, the sequenced pair-end reads were mapped onto the assembled transcriptome, and the read count for each gene was obtained from the mapping results. Mapped reads were used for quantification by RSEM software [45]. Gene or isoform abundance was represented by the fragment per kilobase of transcript per million fragment mapped (FPKM) value, and those transcripts with FPKM values equal to or larger than 0.3 were defined as expressed. Differential expression analysis of two treatments was performed using the DEGseq R package [46]. Three independent biological replicates for each treatment were analysed. The *P* value was adjusted using a q value [47]. Q value < 0.005 was set as the threshold for significantly different expression.

### Validation of AS events by qRT-PCR

qPCR assays were performed to confirm the AS events. 5 μ g of total RNA was treated with 10 U of DNase I (Thermo Scientific, USA) to remove residual DNA and then used for reverse transcription with TransScript First-Strand cDNA Synthesis Super Mix (TransGen). qRT-PCR was performed as described previously [48]. Three independent biological replicates for each sample and three technical replicates for each biological replicate were analyzed. The *P. giganteum* ACT gene was used as the internal control. All of the primers that were used are listed in Additional file 10: Table S7.

## Supplementary information

**Additional file 1 Figure S1.** The subreads distribution (a) and the Flnc reads distribution (b) of *P. giganteum* transcriptomes.

**Additional file 2 Figure S2.** Different splicing isoforms of the same UniTransModels in RT and CT samples. For each isoform, blocks in blue represent exons and lines in- between represent introns.

**Additional file 3 Figure S3.** Venn diagram of differentially expressed genes during cold stress.

**Additional file 4 Table S1.** Characteristic of output data of SMRT sequencing.

**Additional file 5 Table S2.** Distribution of transcripts and unigenes.

**Additional file 6 Table S3.** Summary of unigenes annotation.

**Additional file 7 Table S4.** Transcription Factors identification.

**Additional file 8 Table S5.** LncRNA prediction.

**Additional file 9 Table S6.** UniTransModels had AS events involved in α-linolenic acid biosynthesis and metabolism pathways.

**Additional file 10 Table S7.** List of primers used in qRT-PCR assays.

## Data Availability

All the data was available in present paper. The sequencing data was available at NCBI database with accession number of PRJNA544770.

## Abbreviations

CCS: Circular Consensus Sequencing
CNCI: Coding-Non-Coding-Index
COG: Cluster of Orthologous Groups
Cogent: Coding GENome reconstruction Tool
CPC: Coding Potential Calculator
CT: Chilling Temperature
DEG: Differentially expressed genes
Flnc: Full-length non-chimeric
Go: Gene Ontology
Iso-Seq: Isoform Sequencing
KEGG: Kyoto Encyclopedia of Genes and Genomes
lncRNA: Long non-coding RNA
Nr: NCBI non-redundant protein sequences
Nt: NCBI non-redundant nucleotide sequences
PLEK: Predictor of Long non-coding RNAs and Messenger RNAs based on an Improved k-mer Scheme
RT: Room Temperature
SMRT: Single Molecule Real-Time
SSR: Simple Sequence Repeat
TAs: Trienoic fatty Acids
TF: Transcription Factor
