# Supplementary figures and images for: SMRT sequencing of a full-length transcriptome reveals transcript variants involved in C18 unsaturated fatty acid biosynthesis and metabolism pathways at chilling temperature in Pennisetum giganteum
Source: BMC Genomics. 2020 Jan 16;21:52. doi: 10.1186/s12864-019-6441-3 (PMC6966868; doi:10.1186/s12864-019-6441-3)

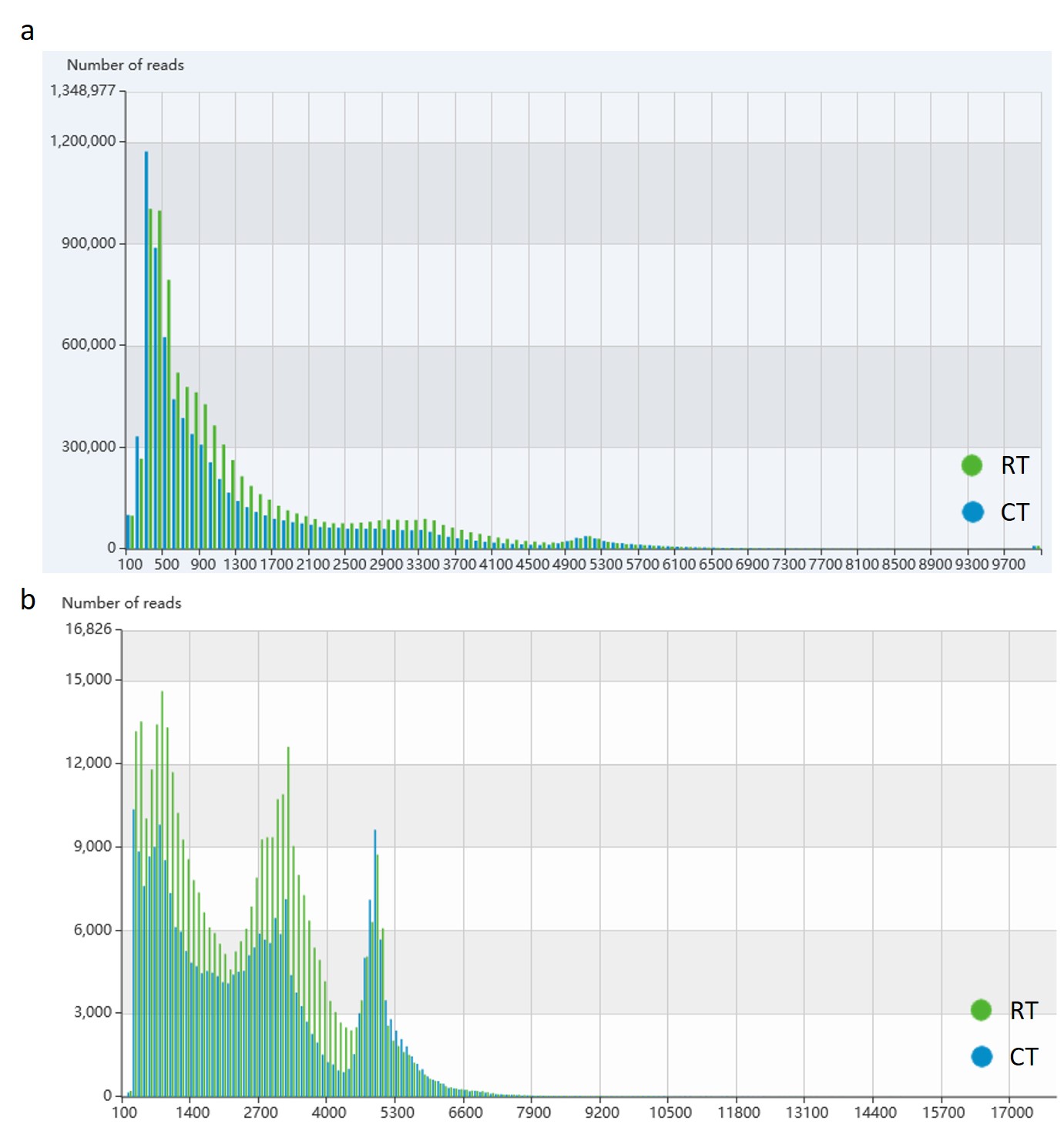

Supplement: Supplementary file 1 — Additional file 1 Figure S1. The subreads distribution (a) and the Flnc reads distribution (b) of P. giganteum transcriptomes. [file 12864_2019_6441_MOESM1_ESM.jpg]

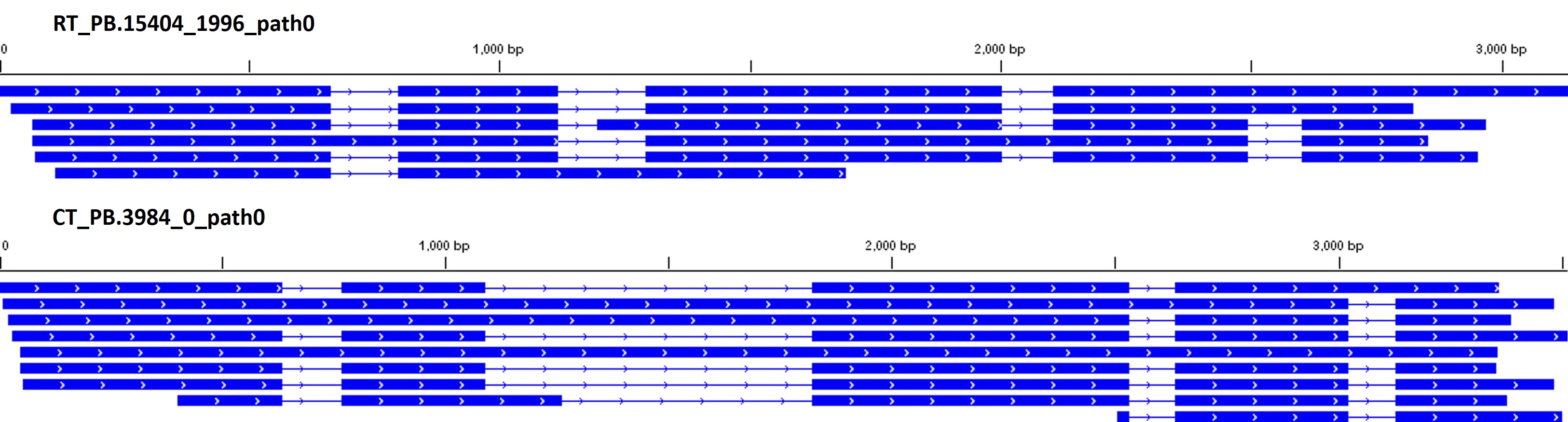

Supplement: Supplementary file 2 — Additional file 2 Figure S2. Different splicing isoforms of the same UniTransModels in RT and CT samples. For each isoform, blocks in blue represent exons and lines in- between represent introns. [file 12864_2019_6441_MOESM2_ESM.jpg]

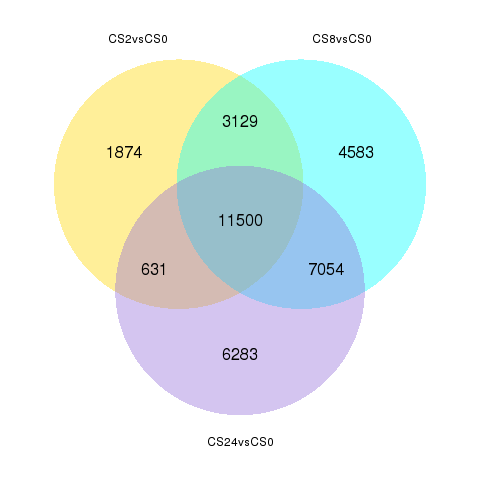

Supplement: Supplementary file 3 — Additional file 3 Figure S3. Venn diagram of differentially expressed genes during cold stress. [file 12864_2019_6441_MOESM3_ESM.png]
